# Analyzing the simplicial decomposition of spatial protein structures

**DOI:** 10.1186/1471-2105-9-S1-S11

**Published:** 2008-02-13

**Authors:** Rafael Ördög, Zoltán Szabadka, Vince Grolmusz

**Affiliations:** 1 Protein Information Technology Group, Department of Computer Science, Eötvös University, Pázmány P. stny. 1/C, H-1117 Budapest, Hungary; 2 Uratim Ltd. Sóstói út 31/b, H-4400 Nyíregyháza, Hungary

## Abstract

**Background:**

The fast growing Protein Data Bank contains the three-dimensional description of more than 45000 protein- and nucleic-acid structures today. The large majority of the data in the PDB are measured by X-ray crystallography by thousands of researchers in millions of work-hours. Unfortunately, lots of structural errors, bad labels, missing atoms, falsely identified chains and groups make dificult the automated processing of this treasury of structural biological data.

**Results:**

After we performed a rigorous re-structuring of the whole PDB on graph-theoretical basis, we created the RS-PDB (Rich-Structure PDB) database. Using this cleaned and repaired database, we defined simplicial complexes on the heavy-atoms of the PDB, and analyzed the tetrahedra for geometric properties.

**Conclusion:**

We have found surprisingly characteristic differences between simplices with atomic vertices of different types, and between the atomic neighborhoods – described also by simplices – of different ligand atoms in proteins.

## Background

The information stored in the Protein Data Bank [1] would make possible fully automated *in silico *studies if mislabeled chemical groups, broken protein- and nucleic acid chains and other errors were corrected. Even today, the newly submitted data is verified "by hand" by human experts. In an earlier work, we applied a rigorous cleaning and re-structuring procedure for the entries in the Protein Data Bank [2], and created the RS-PDB database. We made use of non-trivial mathematical, mainly graph-algorithms: Computing the InChI™ code [3,4] applied a graph-isomorphism testing, transforming aromatic notation to Kekule-notation used a non-bipartite graph-matching algorithm [5], breadth-first-search graph traversals [6] were used throughout the work [2], depth-first search [6] was used in building the ligand molecules and identifying ring structures, kd-trees [7] were applied for computing covalent bonds, and hashing [6] were utilized for the fast generation of protein-sequence ID's.

The resulting RS-PDB database is capable to serve intricate structural queries on all the three-dimensional protein structures known to mankind.

It is of basic importance to map the physico-chemical properties of protein-ligand binding sites, most importantly the Coulomb and Van der Waals forces, in order to predict protein-ligand binding, to design ligands for a given binding site on the surface on a protein, or in designing inhibitors or activators in enzymatic mechanisms. The exact description of the forces in question are deep quantum-chemical problems. The atomic environment of the binding sites clearly has strong effect to these forces; consequently, by examining the atomic environments of the ligands in the crystallographically verified protein-ligand complexes in the PDB would yield insight in binding mechanisms and biologically active molecule design. The first step in this direction need to be the analysis of the simplicial structures of the atoms, forming the protein structures themselves. The second step is the analysis of simplicial neighborhoods of the ligand atoms.

In the present work we define a certain simplicial decomposition on the heavy atoms of the protein structures in the PDB, and analyze some geometrical properties of the tetrahedra of different atomic composition. By this way we – first time in the literature – succeeded in defining a structure capable to answer topological questions concerning the distribution of volume and shape of heavy protein-atoms in the whole PDB. One of our main results is the identification of the volume-shape relation of tetrahedra of distinct atomic composition.

### Delaunay-decompositions

Even the refined, cleaned RS-PDB database [2] lacks important features, such as easy acceptance of queries such as: What atoms surround a certain (ligand- or protein-) atom in the structure? Which atoms are neighbouring with the atom/amino acid X in the protein? How many ligand-atoms are surrounded by exactly the tetrahedron with C-C-C-O atoms in its vertices? How frequent are the tetrahedra with vertices C-C-O-N? Are there differences in the shape of tetrahedra of different composition?

Note, that such queries cannot be answered from the amino-acid sequence of the protein, since they intrinsically depend on the tertiary structure of the protein. Consequently, one need to use some cleaned version of the PDB as the initial data.

We have chosen Delaunay decomposition in the discretization of the dataset in the RS-PDB database, since in this "tessellation", the tetrahedra are close to regular ones, and it is a natural and well defined notion, with a well-known algorithm for the generation of the tessellation.

**Definition 1 ***Given a finite set of points A *⊆ *R*^3^, *and a H *⊆ *A such that the points of H are on the surface of a sphere and the sphere does not contain any further points of A, then the convex hull of H is called a Delaunay region*.

Delaunay regions define a partition of the convex hull of *A*. If the points of *A *are in general position, (i.e., no five of the points are on the surface of a sphere), then all regions are tetrahedra.

Singh, Tropsha and Vaisman [8] applied Delaunay decomposition to protein-structures as follows: they selected *A *to be the set of C_*α *_atoms of the protein, and analyzed the relationship between Delaunay regions volume and "tetrahedrality" and amino acid order in order to predict secondary protein structure.

They gave the following definition:

**Definition 2 **([8]) *The tetrahedrality of the tetrahedron with edge-lengths *ℓ_1_, ℓ_2_, ℓ_3_, ℓ_4_, ℓ_5_, ℓ_6 _*is defined*

$$4{\left(\sum_{k}\ell_{k}\right)}^{2}\sum_{i<j}\frac{{\left(\ell_{i}-\ell_{j}\right)}^{2}}{15}$$

*where *ℓ_*i *_*is the length of edge i*.

Note, that the tetrahedrality of the regular tetrahedron is 0.

## Results and discussion

In what follows *A *⊆ *R*^3 ^is always a subset of the atoms of a protein, preferably heavy-atoms (i.e., non-hydrogen atoms) or just the C_*α *_atoms.

To find the Delaunay decomposition of a set, the *qhull *algorithm was used (the implementation source is available at: [9]).

### The test-set

Our complete test set was selected from the RS-PDB by the following criteria: the entry need to contain at least one protein, with no missing atoms, and the resolution of the structure has to be at least 2.2 Å. We have found 5,757 such entries in the RS-PDB database.

**Figure 1:**
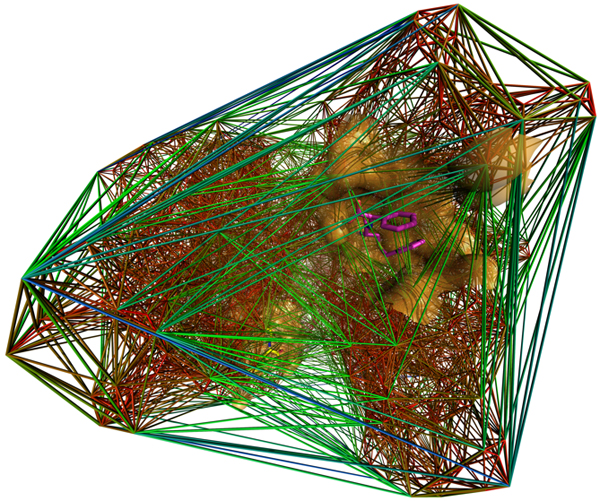
The Delaunay decomposition of the PDB entry 10gs.

Figure 1 shows the decomposition for the PDB entry 10gs.

**Figure 2:**
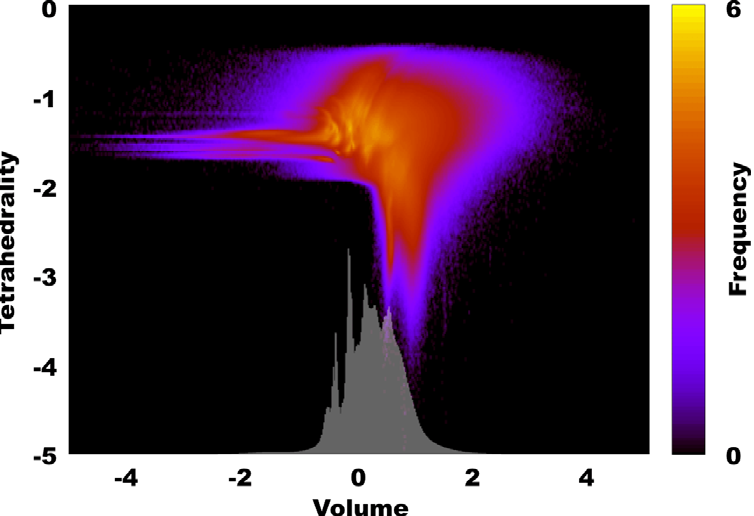
**The triple logarithmic plot of the density of Delaunay regions**. A point with coordinates (*x*, *y*) on the plot corresponds to all Delaunay regions whose volume is 10(*x *± 0.01) and tetrahedrality is 10(*y *± 0.01) and the color of the point corresponds to log(*z *+ 1) where *z *is the number of such regions. The white barplot on the bottom of the image is the same for volume only.

In contrast with the article [8], we have taken *A *to be the set of heavy atoms of the 5757 proteins. Note that in that case we cannot assume that points are in general position, as for example in a (perfect) benzene ring at least 6 carbon atoms lie on a sphere. However, we have found that – probably due to both imprecision of data in the PDB and minor perturbations in atomic positions – all regions are tetrahedra. In our test we – instead of examining the distribution of volume and tetrahedrality of regions separately – created density maps in both variables at the same time. The triple logarithmic plot can be seen on Figure 2. It is quite straightforward to see that at the boundary of the protein the tetrahedra tend to be more irregular and of larger volume, while in the inside of the protein, the tetrahedra are small, compact, and regular (see Figure 1). However, the more intricate analysis depicted on Figure 2 shows a distinctly characteristic distribution. One of our main results is the identification of regions of the plot of Figure 2, strictly characteristic to the vertex-composition of the tetrahedra involved.

### Labeling the vertices of the tetrahedra

After that we examined tetrahedra grouped according to the set of atoms in their vertices. All tetrahedra were assigned a label that is the merging of the 4 symbols assigned with the elements in the corners in alphabetic order. (For example a tetrahedra spanned by a nitrogen, two carbon atoms and an oxygen would be assigned the symbol: C_C_N_O_. Grouped by these labels, we listed the count of the tetrahedra in Table 1.

**Table 1:** The counts of different types of Delaunay tetrahedra in the test set of 5,757 PDB entries. Tetrahedron C_C_N_O_ (containing the peptide bound of amino acids) turns out to be the most frequent with 19, 463, 268 occurences in our test set. The frequency of other labels decrease exponentially.

Pattern	Count	Pattern	Count	Pattern	Count
C C N O	19,463,268	C C C O	13,979,006	C C C N	9,228,670
C C C C	8,549,030	C C O O	8,302,189	C N O O	7,148,317
C N N O	4,811,063	C C N N	4,137,294	C O O O	1,774,801
N N O O	983,656	N O O O	696,899	C C C S	575,423
C C O S	453,511	C N N N	320,021	C C N S	305,453
C N O S	255,407	O O O O	220,453	N N N O	184,983
C O O S	99,173	C C S S	56,480	C N N S	42,572
N O O S	30,644	C O S S	23,276	N N N N	21,076
C N S S	19,843	N N O S	16,119	O O O S	8,380
C C C SE	7,624	N O S S	4,995	C C O SE	4,582
C C N SE	2,822	C N O SE	2,289	N N N S	1,982
N N S S	1,872	C S S S	1,848	O O S S	1,565
N S S S	793	C O O SE	764	C N N SE	433
S S S S	420	O S S S	335	C C C F	256
N O O SE	230	C C F O	224	N N O SE	149
C O O P	145	C C F N	123	O O O P	101
C C SE SE	99	C F N O	96	N O O P	91
C C S SE	72	O O O SE	70	C C C I	65
C C I O	51	C F O O	47	C CL N O	40
C N O P	38	N N N SE	31	C I O O	28
C C CL N	27	C C CLO	26	C O S SE	25
AS C C S	21	AS C C O	20	C I N O	20
C N SE SE	19	AS C C C	17	C F N N	16
C C O P	15	AS C O S	15	C C C CL	15
F N O O	14	C O O V	12	C C I N	12
AS C N O	11	B C O O	10	C CLO O	10
AS C C N	10	C O SE SE	9	C C F S	9
O O O V	8	F N N O	6	C C I S	6
N N O P	6	AS C O O	6	AS N O O	5
C N S SE	5	B C N O	4	N N SE SE	4
B C C O	4	CL N O O	4	I O O O	4
I N O O	4	CL N N O	3	N O SE SE	3
F O O O	3	AS N N O	3	AS C N N	3
N O S SE	3	C I O S	2	C C F F	2
B N O O	2	C O P S	2	C F O S	2
AS C N S	1	O O S SE	1	C F F N	1
C C C P	1	O O P S	1	N N S SE	1
AS O O S	1	AS N O S	1	C CL N N	1

### Volume-shape distribution of different types of tetrahedra

**Figure 3:**
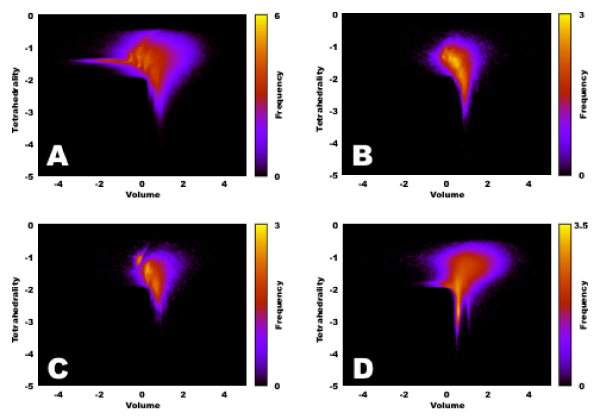
**Separate drawing for different tetrahedra**. We give here similar density maps as in Figure 2, but now separately drawn for tetrahedra with vertices C_C_N_O (inset A), C_C_O_S (inset B), C_N_O_S (inset C) and N_N_O_O (inset D). It is clear that different vertex-compositions implies different shape/volume distributions.

We observed that splitting the density plot according to the composition of the vertex-sets of the Delaunay tetrahedra would show different patterns for different labels. This is one of our main results, depicted on Figure 3.

### Ligand atoms in tetrahedra from proteins

Here we analyze the atomic environments of ligand atoms, bound to proteins. The atomic environment of each ligand atom will be identified as the vertices of a tetrahedron in a tetrahedral decomposition of the heavy atoms of the protein, containing the atom of the bound ligand.

**Figure 4:**
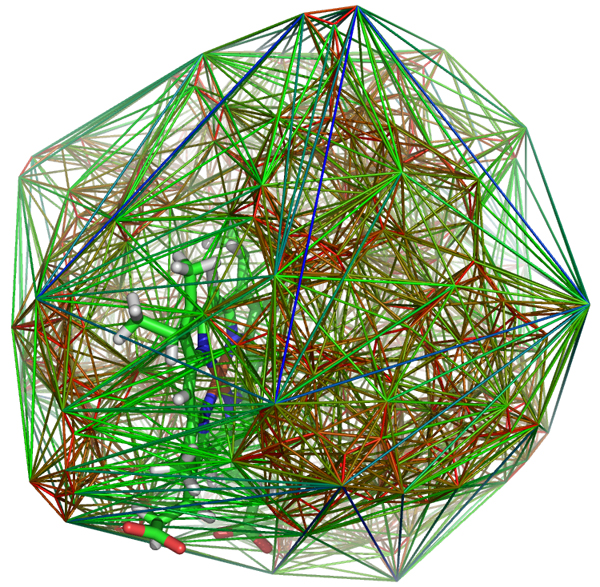
**Ligand in a Delaunay decomposition**. The Delaunay decomposition of the PDB entry 1n9c. The ligand is pictured with solid lines.

By this approach we can describe uniformly and in a discreet manner the environment of ligand atoms in proteins. The classification is given by describing tetrahedra according to the atoms in their vertices, and by the atoms of the ligands the convex hull these tetrahedra contain (Figure 4). One of our main results is the statistical analysis of the frequencies of the separate ligand atoms in different types of tetrahedra, formed from protein atoms in Table 2 and Table 3.

**Table 2:** The classifications of the tetrahedra around metal ligand atoms. The tetrahedra not present contain no metal atoms.

	**CCNO**	**CNOO**	**CNNO**	**COOO**	**CCNN**	**NNOO**	**NOOO**	**CNNN**
**Zn**	5	7	3	2	0	14	14	0
**Mg**	1	11	4	1	0	15	10	6
**Ca**	0	0	0	0	0	0	1	0
**Mn**	0	0	0	3	1	3	4	0
**Fe**	2	3	0	1	1	0	0	2

	**NNNO**	**OOOO**	**CCCS**	**NNNN**	**CCNS**	**CCSS**	**NOSS**	

**Zn**	38	2	0	0	4	0	32	
**Mg**	5	6	0	5	0	0	0	
**Ca**	2	48	0	0	0	0	0	
**Mn**	4	5	0	0	0	0	0	
**Fe**	0	0	2	1	0	5	0	

**Table 3:** The classifications of the tetrahedra around frequent non-metallic ligand atoms. An atom is called frequent, if it appears in at least 100 entries in our data set.

	C C N O	C C C O	C C O O	C N O O	C C C C	C N N O	C O O O	C C C N	C C N N	N N O O	N O O O
H	5590	5385	5461	4678	3091	2651	2899	2360	1304	1328	1334
C	4218	4289	3757	3295	2628	1806	1777	2091	1125	839	886
O	1673	823	1097	1470	345	1373	621	601	623	731	519
N	585	554	589	605	195	220	447	307	97	150	187
P	41	10	17	30	6	110	17	18	38	64	28
S	77	42	43	49	27	31	16	28	21	9	9
F	27	40	42	22	31	14	6	18	5	5	2

	C N N N	N N N O	O O O O	C C O S	C C C S	N N N N	C N O S	C O O S	C C N S	N N O S	N O O S

H	663	583	665	325	298	139	204	187	149	88	66
C	422	317	276	226	267	70	132	107	133	50	32
O	524	521	133	47	41	214	92	37	45	31	20
N	36	40	170	56	29	6	39	33	19	7	7
P	70	75	4	1	0	69	0	0	0	0	1
S	6	5	3	9	5	1	4	4	1	1	0
F	0	2	0	0	2	0	1	0	0	0	0

	C N N S	C C S S	N O S S	O O O S	C N S S	N N N S	O O S S	N N S S	C O S S	C N O S E	C N N S E

H	32	16	2	18	19	5	7	9	7	1	1
C	30	59	3	11	11	9	7	2	2	0	0
O	29	8	8	3	1	7	0	4	2	0	0
N	4	0	2	2	0	0	7	0	3	0	0
P	2	0	0	0	0	0	0	0	0	0	0
S	0	0	0	0	0	0	0	0	0	2	2
F	0	0	0	0	0	0	0	0	0	0	0

### Identifying ligands

We are using the ligand-identification technique described in [2], using the classification of monomer ID's given in [10] and [11]. Concisely, we doubly checked if a ligand, even with more than one monomer ID's is one molecule or not, by comparing the bond tables from mmCIF and the atomic distances. The ligand was thrown out if recognized as a crystallization artifact, covalently bound (but non-protein-) or junk molecule [10].

## Conclusion

In this work we prepared the simplicial decomposition of 5,757 protein structures, chosen from the Protein Data Bank by quality criteria such as every atom has coordinate (i.e., there are no missing atoms) and the resolution of the structure is at least 2.2 Å. The heavy atoms (that is, non-hydrogen atoms) of the structures were decomposed into Delaunay regions using the *qhull *algorithm [9]. Next we depicted the tetrahedrality/volume relation in a triple logarithmic plot (Figure 2), and also counted the tetrahedra of different vertex-sets in Table 1. We found that tetrahedra with different atoms in their vertices populate different areas of the plot of Figure 2: Figure 3 gave our results. Figure 3 shows, that data-points, corresponding to tetrahedra of a given atomic composition assume well-characterizable positions in Figure 2. This result show the spatial preferences in tetrahedra of distinct composition in protein structures. By further exploring this avenue methods may appear in helping *in silico *protein folding studies. We also used the RS-PDB database [2] for finding crystallographically verified ligands in our test-set of 5,757 proteins. Next the tetrahedra, containing the atoms of these ligands were collected and given in Tables 2 and 3. We believe that these large-scale data will help in *in silico *identifying ligand-binding preferences in inhibitor design and in ligand binding prediction.

## Competing interests

The authors declare that they have no competing interests.

## Authors' contributions

Rafael Ördög designed and prepared the simplicial database, analyzed it with the triple-logarithmic plots of Figure 2, and Figure 3, and analyzed the data of tetrahedra of different atomic types and ligands. Zoltán Szabadka designed and prepared the RS-PDB database, including the cleaning methods, and helped the discretization. Vince Grolmusz initiated the simplicial decomposition of the protein spatial data, lead the work and wrote the paper.
